# Unravelling the Mystery of a Continuous Coil: A Case Report

**DOI:** 10.21980/J8PM00

**Published:** 2022-04-15

**Authors:** Ryan Brown, Sharon Kim, Robert Tennill

**Affiliations:** * Southern Illinois University School of Medicine, Department of Emergency Medicine, Springfield, IL; ^ Southern Illinois University School of Medicine, Center for Clinical Research, Springfield, IL

## Abstract

Endovascular coil embolization continues to become a more frequent modality of addressing hemorrhage and bleeding in patients. Migration refers to the coil unraveling or moving from the original embolization site. Migration of a coil is a known complication which can lead to serious consequences based on where the coil migrates. Despite increasing efforts to improve safety and technique, the risk of migration remains. We present a case of an embolization coil that migrated to the right ventricle, which was incidentally found roughly 2 months after undergoing an interventional radiology procedure for gastric variceal bleeding. The patient presented to the emergency department with dyspnea and abdominal pain. Unique images were obtained during his visit and in subsequent follow-up. As use of vascular embolization coils continues to become more commonplace, understanding the risks and complications of these procedures remains an important aspect of providing care for patients once they have left the interventional radiology suite. Coil migration should be a differential to consider in patients who present to the emergency department with signs or symptoms of arrhythmia or pulmonary embolism who have undergone a coil embolization procedure.

**Topics:**

Case report, coil embolization, radiology.

**Figure:**
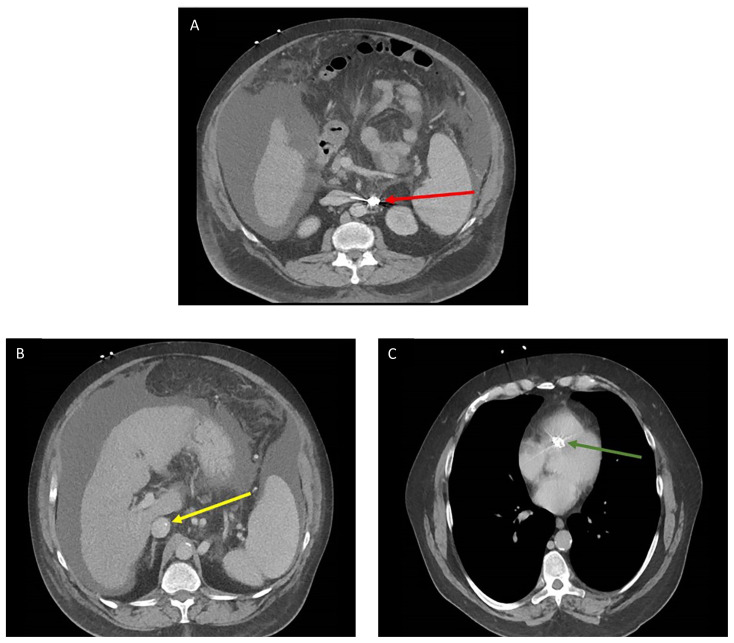


**Figure:**
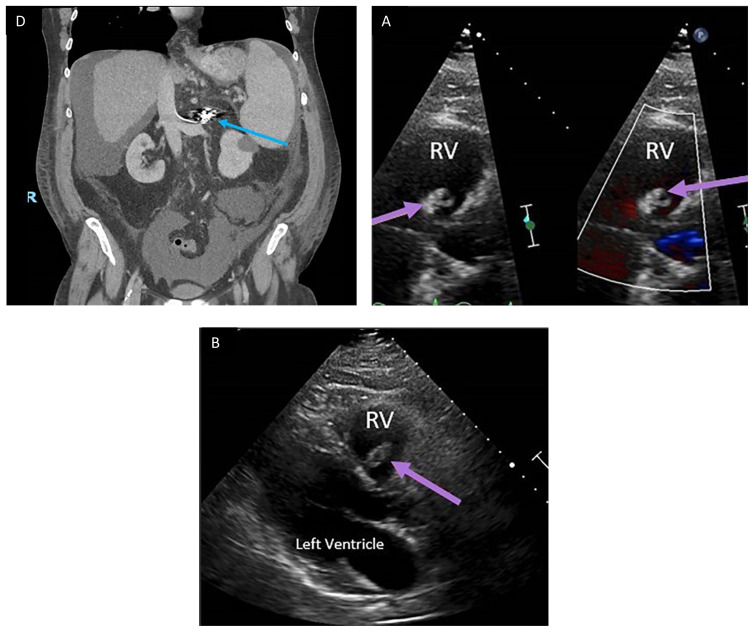


**Figure:**
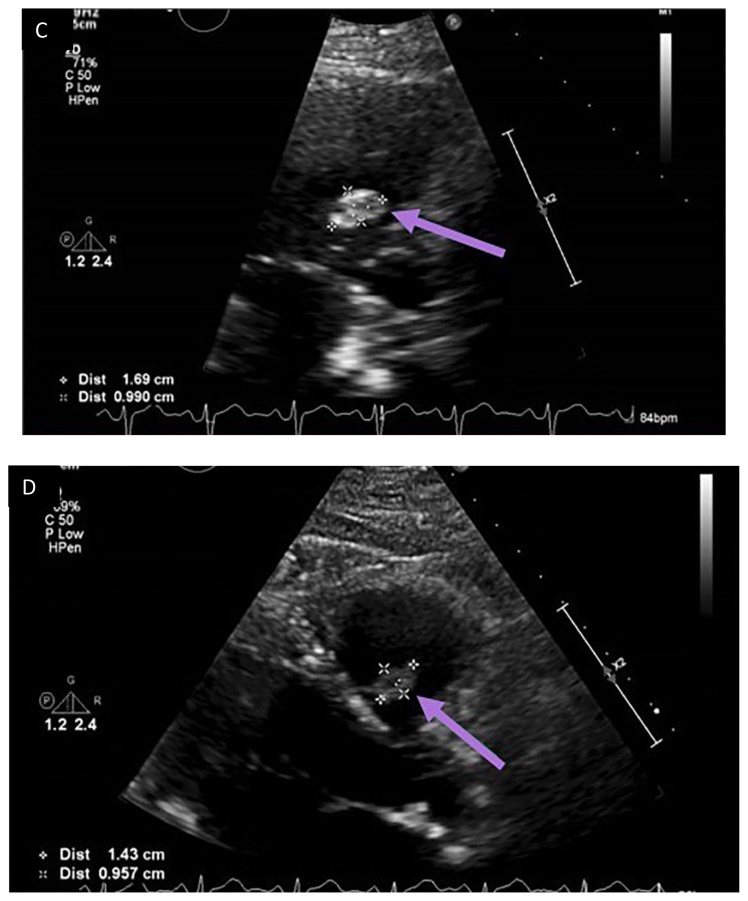


## Brief introduction

Since its inception, the use of endovascular coil embolization has become a common intervention for patients who present with hemorrhage. This minimally invasive technique allows for direct occlusion of a vessel and is a relatively low risk procedure when compared to other methods of internal hemorrhage control. Despite increasing improvements in safety and technique, complications may still arise. Notably, migration of the coil is a known complication which can occur despite utilizing various methods to ensure that the coil remains secure in the intended location.^1^–^5^ In this case report, we will discuss a patient who underwent a coil-assisted retrograde transvenous obliteration (CARTO) procedure for gastric varices who later presented to the emergency department with dyspnea and abdominal pain and was found to have unraveling of the coil into the right ventricle. This particular complication is rare and could provide insight into how to approach post-procedural imaging and follow-up when coils unravel following the procedure. It also provides a new differential diagnosis for emergency medicine providers to consider when a patient presents with signs or symptoms of arrhythmia or pulmonary embolism in a patient who has had prior vascular coil embolization.

## Presenting concerns and clinical findings

A 59-year-old male with a history of cirrhosis, alcohol abuse, and gastrointestinal bleeding presented to the emergency department with one week of abdominal pain and distension. This was associated with dyspnea, nausea, and a 7-kilogram weight gain as well. The patient denied any fever, chills, or recent illnesses. Remaining review of systems was non-significant. Vital signs showed a temperature of 36.7 degrees Celsius, blood pressure of 115/66 millimeters of mercury, heart rate of 90 beats per minute (bpm), respiratory rate of 18 breaths a minute, and an oxygen saturation of 94% on room air. The patient was alert and oriented to person, place, and time.

Physical examination revealed a large, distended abdomen with tenderness to palpation of the lower quadrants and epigastric area. There was no asterixis. There was an overlying area of erythema and edema in the lower quadrants as well. The patient had multiple telangiectasias noted throughout the skin. Subsequent labs were notable for hemoglobin of 12.5 grams per deciliter (g/dL) (reference range: 14–17 g/dL), white blood cell count of 3.4 x10^9^ L (reference range: 3.2–9.8 x10^9^/L), alkaline phosphatase of 172 international units per liter (IU/L) (reference range: 36–92 IU/L), aspartate aminotransferase of 40 IU/L (reference range 13–39 IU/L), alanine aminotransferase of 20 IU/L (reference range 7–52 IU/L), direct bilirubin of 1.0 milligrams per deciliter (mg/dL) (reference range: 0.0–0.3 mg/dL), total bilirubin of 2.4 (mg/dL) (reference range: 0.3–1.2 mg/dL), and high urobilinogen in the urinalysis. Electrocardiogram showed normal sinus rhythm with a heart rate of 82 bpm and no acute abnormalities or ischemic changes. A one-view anterior-posterior X-ray of the chest did not show any acute processes to explain his symptoms. Additional imaging with a computed tomography (CT) scan of the abdomen and pelvis was also obtained which will be discussed below in the “Significant Findings” section.

Upon review of previous records, it was found that roughly two months prior the patient had presented to the same hospital with a five-day history of epigastric abdominal pain and melena. At that time, he was severely hypotensive and anemic with a hemoglobin of 8.1 g/dL, which had been 13.7 g/dL 3 months prior. Subsequent esophagogastroduodenoscopy showed new gastric varices thought to be the cause of his presentation. Interventional radiology was consulted to perform the CARTO procedure using an approach through the patient’s splenorenal shunt. Following successful coil embolization of the gastric varix, the coil had unraveled into the patient’s right atrium, and retrieval of the free-floating end was attempted. During the retrieval from the right atrium, the coil fractured and was retrieved with no coil in the right atrium remaining. The remaining end of the coil had a roughly 2.5 centimeter (cm) length unraveled in the cephalad aspect of the left renal vein. This unraveled end was still attached to the coil pack. Contrast was injected into the vein, and it was determined that it was low risk for embolizing the vessel. The patient was asymptomatic following the procedure until he arrived at the emergency department two months later, where the case report resumes.

## Significant findings

A CT scan of the abdomen and pelvis with intravenous contrast for evaluation of new onset abdominal pain and distension was obtained in the emergency department. The axial view (CT Image A) shows the coil pack from the prior coil-assisted retrograde transvenous obliteration procedure, seen in the left renal vein and gastric varix (red arrow). The path of the coil (yellow arrow) is continuous into the inferior vena cava (CT Image B). It is then seen (CT Image C) situated in the right ventricle (green arrow). Finally, the coil pack is seen in a coronal section, demonstrating its upward path (blue arrow) in the inferior vena cava. (CT Image D). Additional findings included ascites with advanced cirrhosis. As noted in the CT images, a vascular embolization coil was seen within a varix near the junction of the left renal vein. This appeared to have unraveled and extended superiorly into the inferior vena cava and ultimately into the right atrium and right ventricle.

Outpatient transthoracic echocardiogram performed in follow-up at a later date after the patient’s emergency department visit is shown above (Echo Images A–D). The images obtained showed a hyperechoic structure (purple arrows) located on the septal leaflet of the right ventricular (RV) side of the tricuspid valve (Echo Images A–D). It measured 1.69 centimeters (cm) by 0.99 cm and moved with valvular motion. There was no reverberation artifact seen on examination. The differential for this included thrombus, bacterial vegetation, myxoma, and coil with overlying fibrous tissue. Given the patient’s recent history, this is likely the coil seen on the CT scan of the abdomen and pelvis obtained previously in the emergency department.

## Patient course

With discovery of the coil in the right ventricle on CT, the emergency department physician was contacted by the diagnostic radiologist who recommended a consultation to interventional radiology for further evaluation and management. The patient was placed on continuous cardiac telemetry and admitted to the hospital for planned interventional procedures of paracentesis and coil retrieval. The patient first underwent ultrasound guided diagnostic and therapeutic paracentesis. Roughly 8 liters of fluid was removed. There was no evidence of spontaneous bacterial peritonitis on evaluation of the ascitic fluid in lab.

Following the paracentesis, the patient was brought to the catheterization suite and underwent fluoroscopy of the coil pack and unraveled end. The motion of the coil in the right heart was synchronous with contraction of the heart. Thus it was determined that based on the imaging obtained, the coil was likely embedded in the cardiac tissue or surrounded by scar tissue. Given the timing, this coil may have migrated to his right heart shortly after or during the CARTO procedure two months prior. Based on these findings, the decision was made to leave the coil in place and arrange for follow-up echocardiogram in four weeks, the results of which are shown above (echo Images A–D). He was discharged from the hospital the next day after an uneventful overnight stay on the general floor.

During his follow-up visit, he denied any new symptoms; notably, there were no palpitations, dyspnea, chest pain, or syncope reported. A hyperechoic structure on the ventricular side of the tricuspid valve measuring 1.69 cm by 0.99 cm in size was seen on echocardiogram (purple arrows in Echo Images A-D). The differential included foreign body migration, vegetation, tumor, and thrombus. This structure appeared to move with the tricuspid valve motion. There was no reverberation artifact typically seen with metal objects on ultrasound. So, it is not definitive whether this was the migrated coil; however, it is likely based on his recent history. The lack of reverberation artifact may have been due to scar tissue or thrombus surrounding the coil. Ultimately it was decided that since the patient was asymptomatic at the time of the visit, he would be monitored with follow-up visits and repeat transthoracic echocardiogram if symptoms were to arise. Thus far, the patient has not had any issues with further coil migration and it remains in stable position on repeat outpatient echocardiograms.

## Discussion

Coil migration to the heart, following transcatheter micro-coil embolization of a vessel, is an uncommon but important complication to consider when performing the procedure or evaluating the patient at a later time. Review of the literature showed that the risk of migration to the heart is dependent on the location and indication for embolization, notably when performing coil embolization of a pulmonary arteriovenous malformation where the incidence of migration is much higher.^3^,^4^ The authors of this paper were unable to find a similar instance of coil embolization to the heart occurring during the CARTO procedure.

A case report by Padiyath et al described the uncoiling of a fractured guidewire during central line placement in an infant. It was suggested that the uncoiling rendered the wire radiolucent and therefore was difficult to see on fluoroscopy. Using transesophageal echocardiogram (TEE), they were able to retrieve the unraveled guidewire.^1^ It is possible that in our case the fracturing of the coil led to the appearance of complete retrieval from the right atrium during the patient’s initial CARTO procedure. Perhaps TEE could be used as an adjunct modality for visualization of migrated wire or coil when fracture is seen or suspected during a procedure. Additional modalities include X-ray, fluoroscopy, and CT, which can be helpful in localizing coil migration.^2^

Regarding management, one study reported that even with high technical skill and experience, retrieval of the coil through percutaneous intervention is only successful roughly 50% of the time.^3^ Risks of percutaneous removal include further migration to the pulmonary circulation, stroke, induced arrhythmia, cardiac tissue perforation, and tamponade. Other methods of retrieval include a thoracotomy and sternotomy to perform open-heart surgery, which is considerably more invasive and has a multitude of complications that can occur in recovery. This was high risk in our patient, especially when compared to the benefit from attempting to intervene in an asymptomatic patient. According to Leitman et al, in a study of foreign bodies in the heart, roughly 54% of the 104 cases underwent surgical intervention for removal. Percutaneous retrieval was done in 29% of patients in the study. The remaining 14% were managed conservatively.^4^ In our patient, the coil appeared to have been surrounded by fibrous tissue on echocardiography. This implies the possibility that the coil is somewhat secured in place and that any attempt to remove it may cause damage to the surrounding tissue.

Finally, this case highlights several important points regarding the common endovascular interventions that have increased in both use and safety since their inception. Periprocedural management of coil fracture should include the application of proper techniques to ensure coil retrieval. Several other cases discussed the need for proper catheter size and snare devices to maximize the chances of successful retrieval of the coil. When complete retrieval is uncertain or migration is suspected, adjunctive imaging modalities should be used to guide further management. Symptoms and predicted complications may also be considered. For the emergency medicine provider, this complication is a rare but important differential diagnosis to consider if a patient has undergone endovascular coiling and presents with signs or symptoms of arrhythmia or pulmonary embolism.

## Supplementary Information
















