# Supplementary figures and images for: Unravelling the Mystery of a Continuous Coil: A Case Report
Source: J Educ Teach Emerg Med. 2022 Apr 15;7(2):V14–20. doi: 10.21980/J8PM00 (PMC10332746; doi:10.21980/J8PM00)

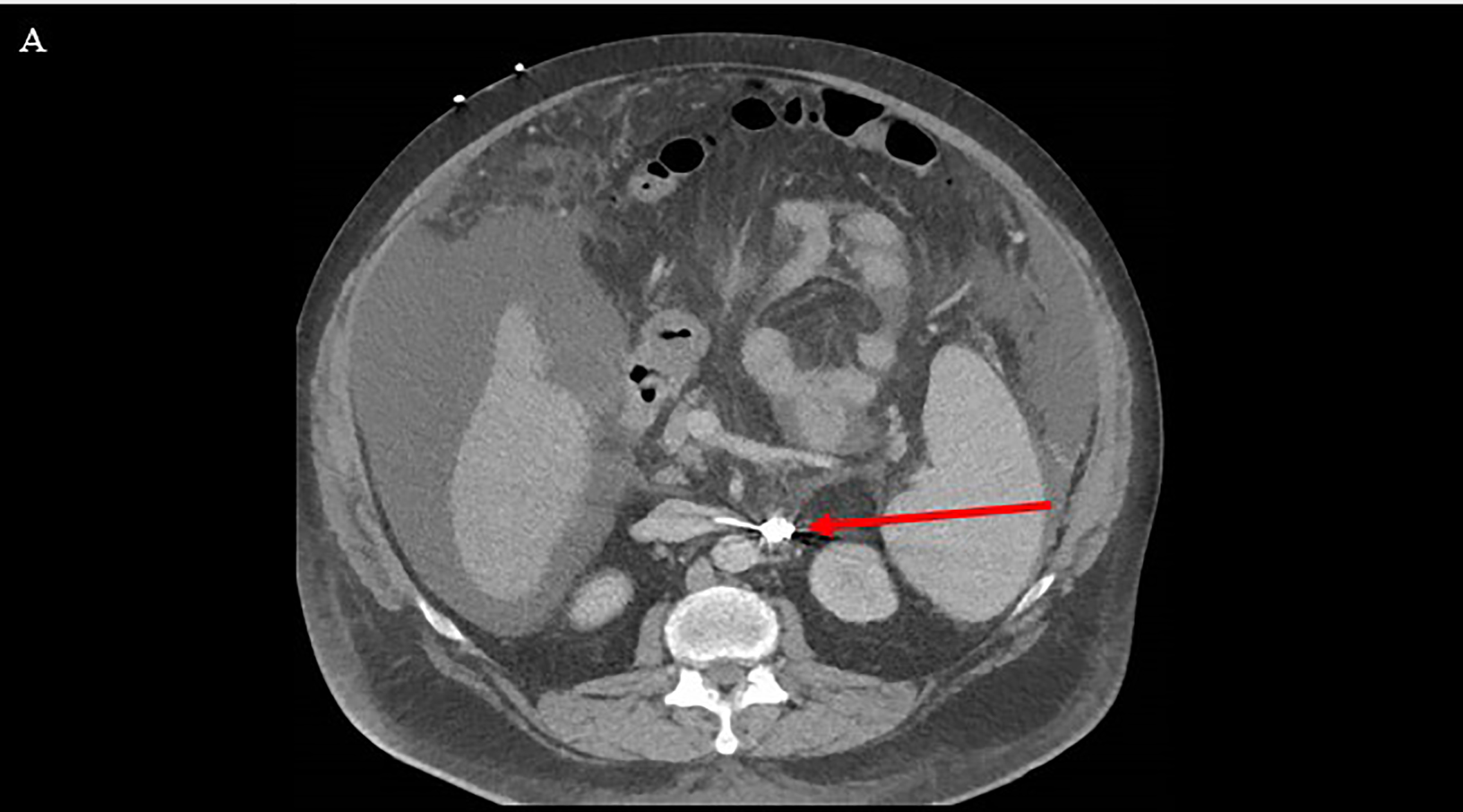

Supplement: Supplementary file 1 [file JETem-7-2-V14supp1.jpg]

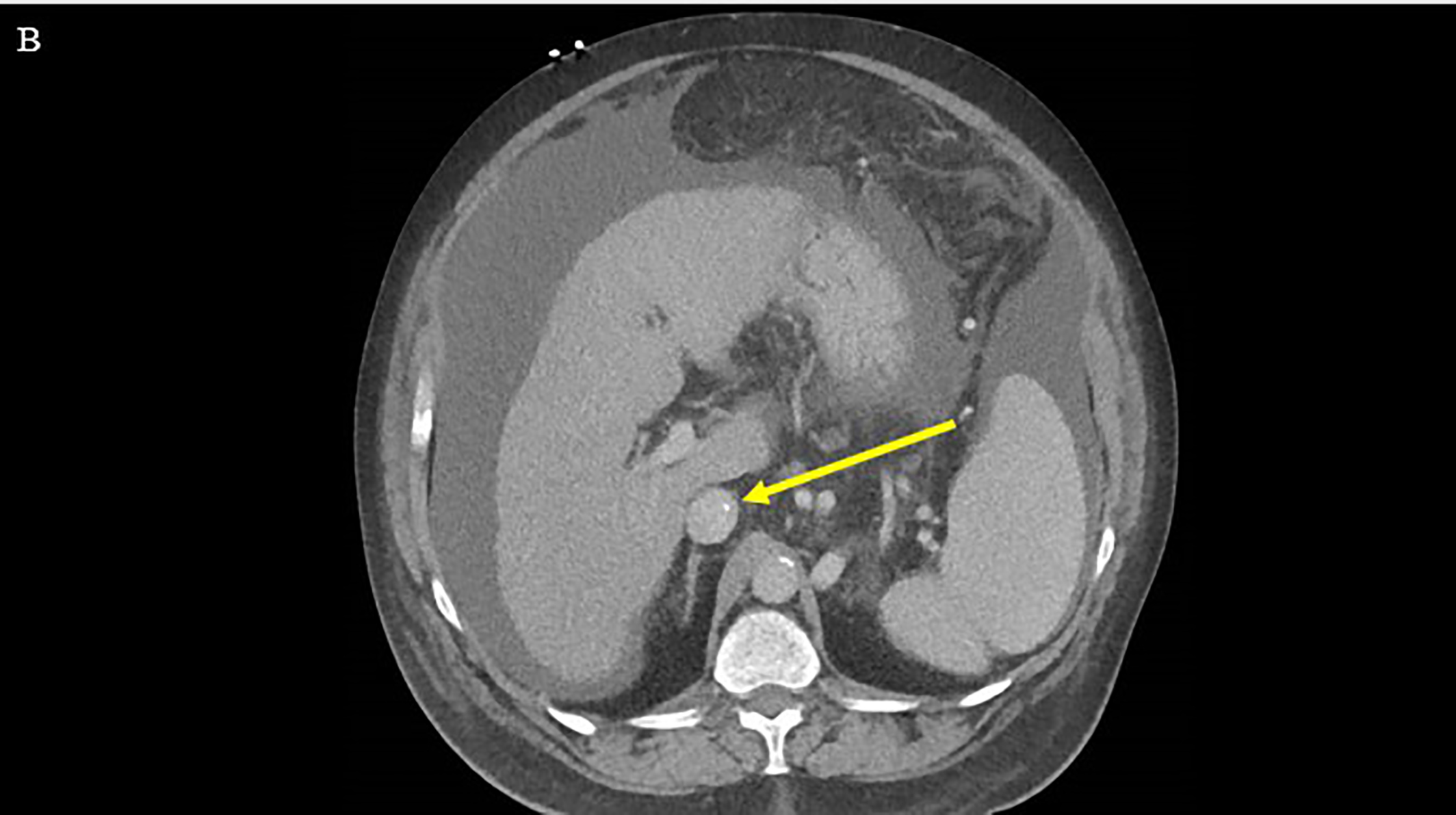

Supplement: Supplementary file 2 [file JETem-7-2-V14supp2.jpg]

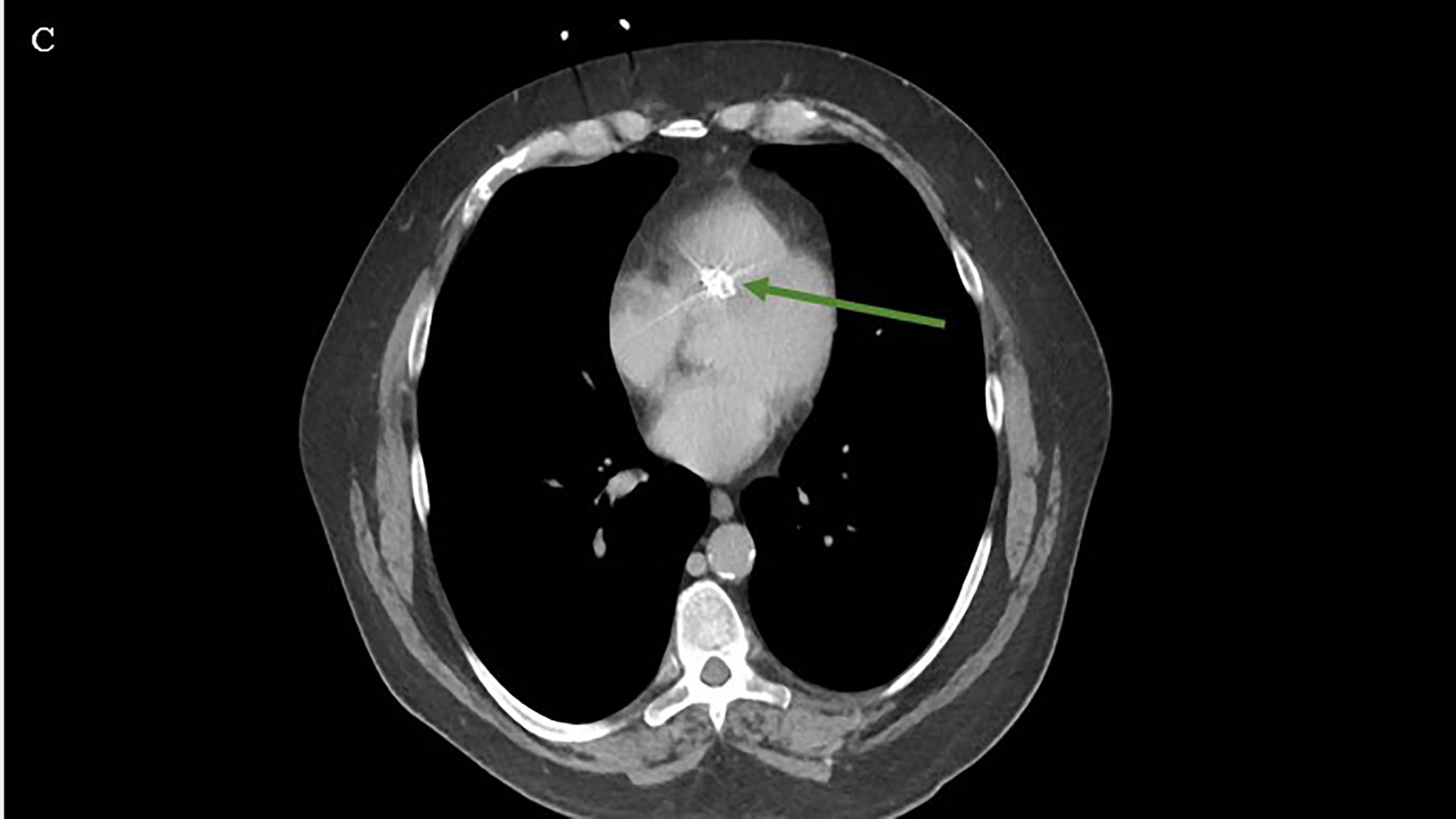

Supplement: Supplementary file 3 [file JETem-7-2-V14supp3.jpg]

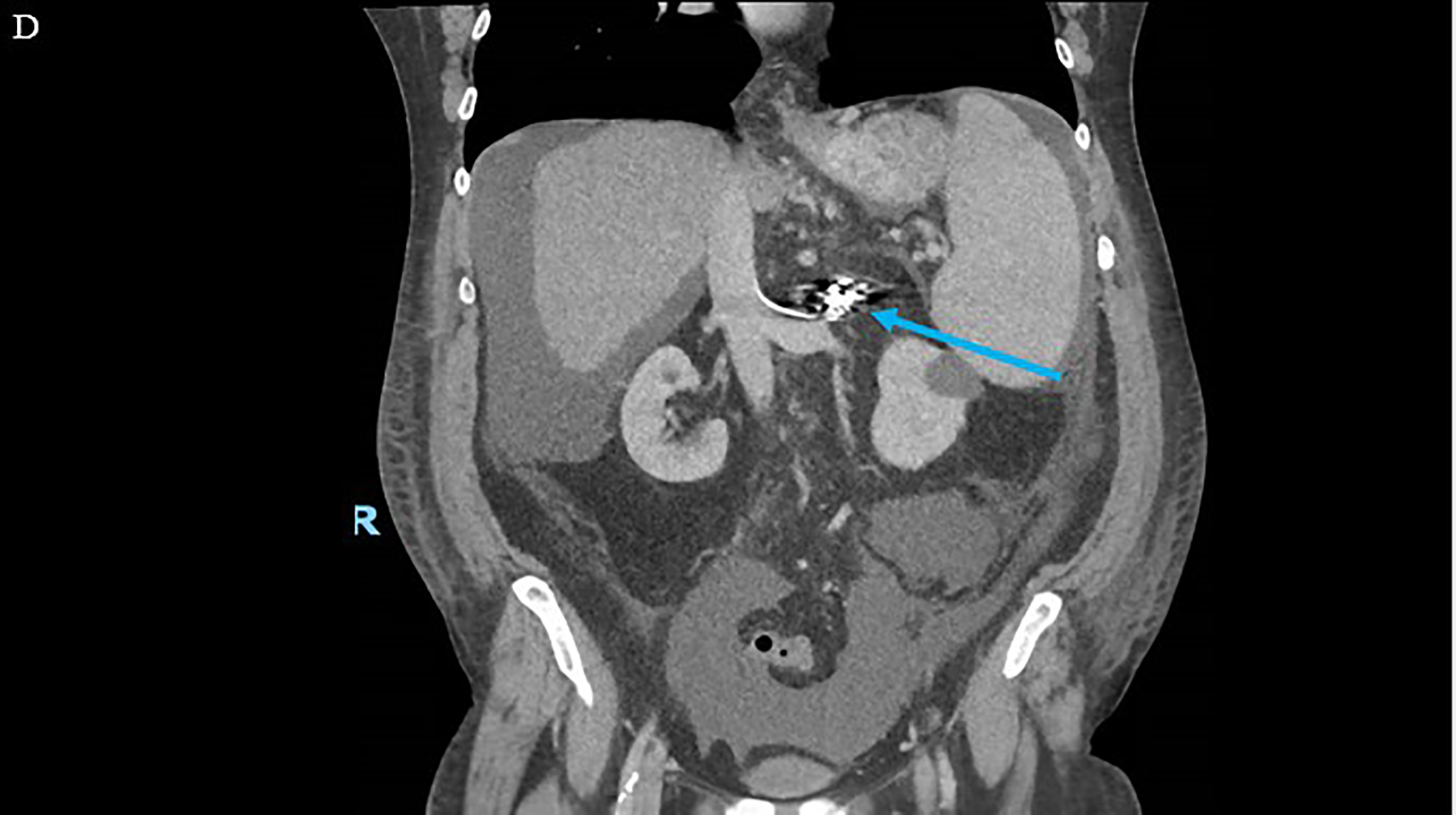

Supplement: Supplementary file 4 [file JETem-7-2-V14supp4.jpg]

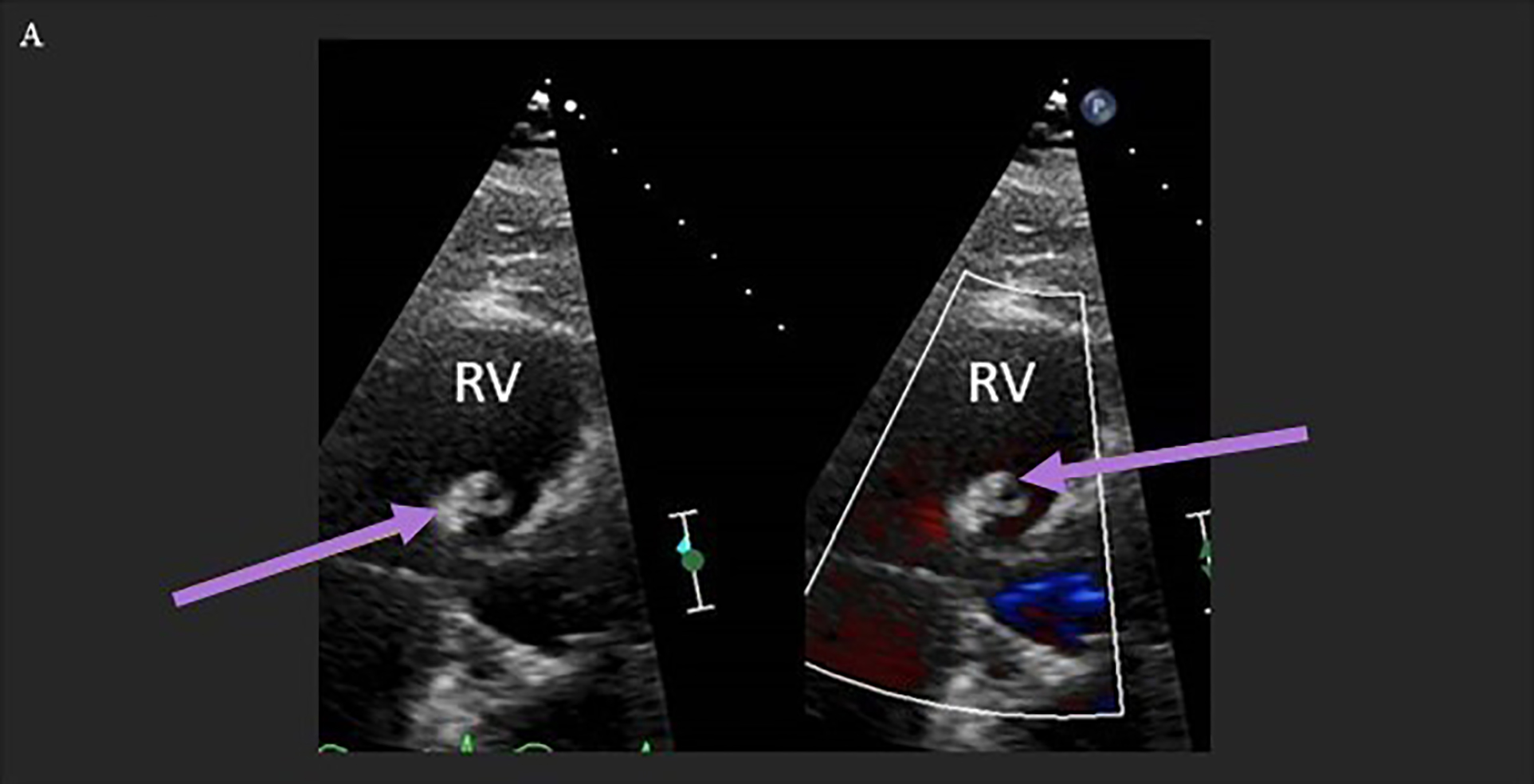

Supplement: Supplementary file 5 [file JETem-7-2-V14supp5.jpg]

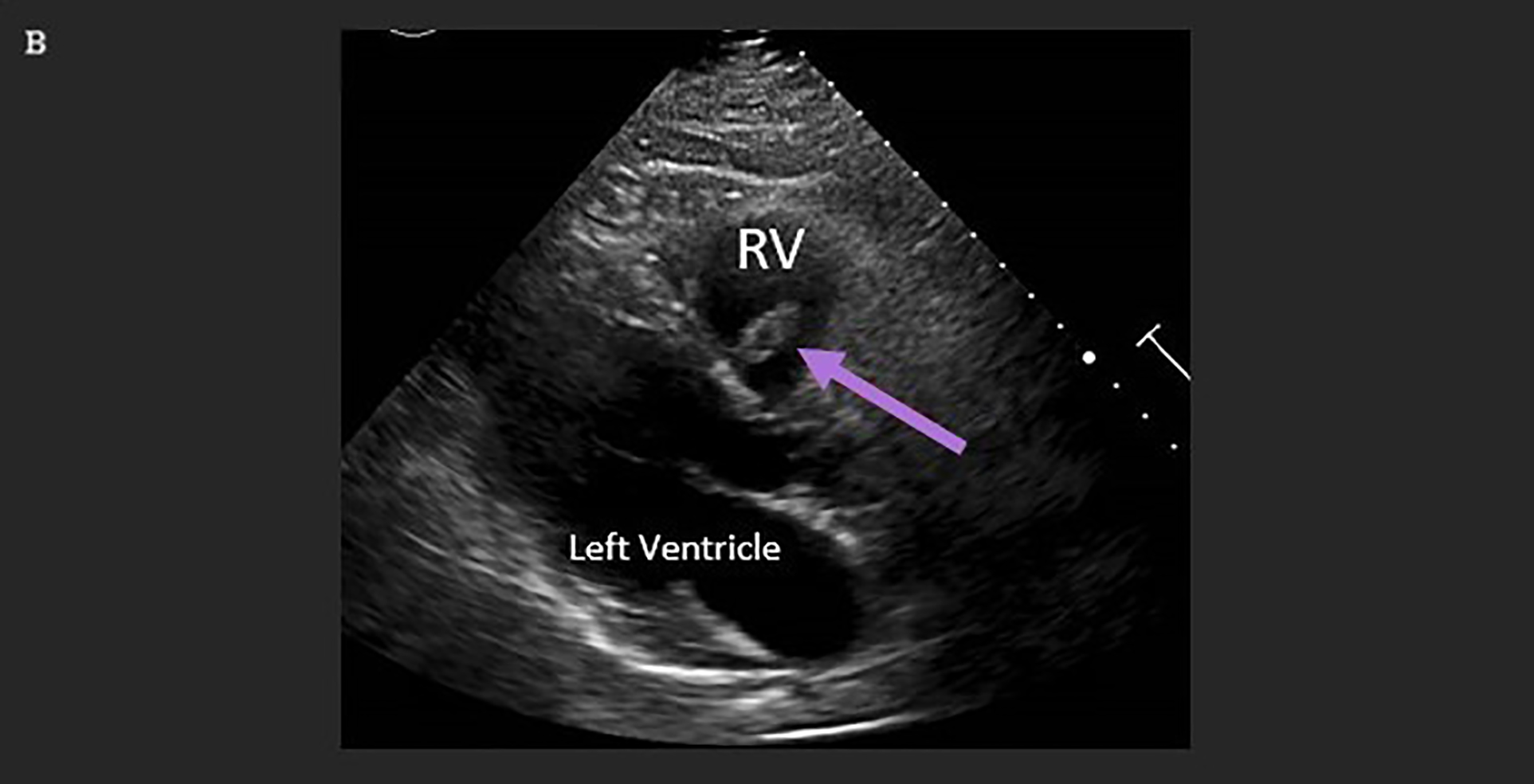

Supplement: Supplementary file 6 [file JETem-7-2-V14supp6.jpg]

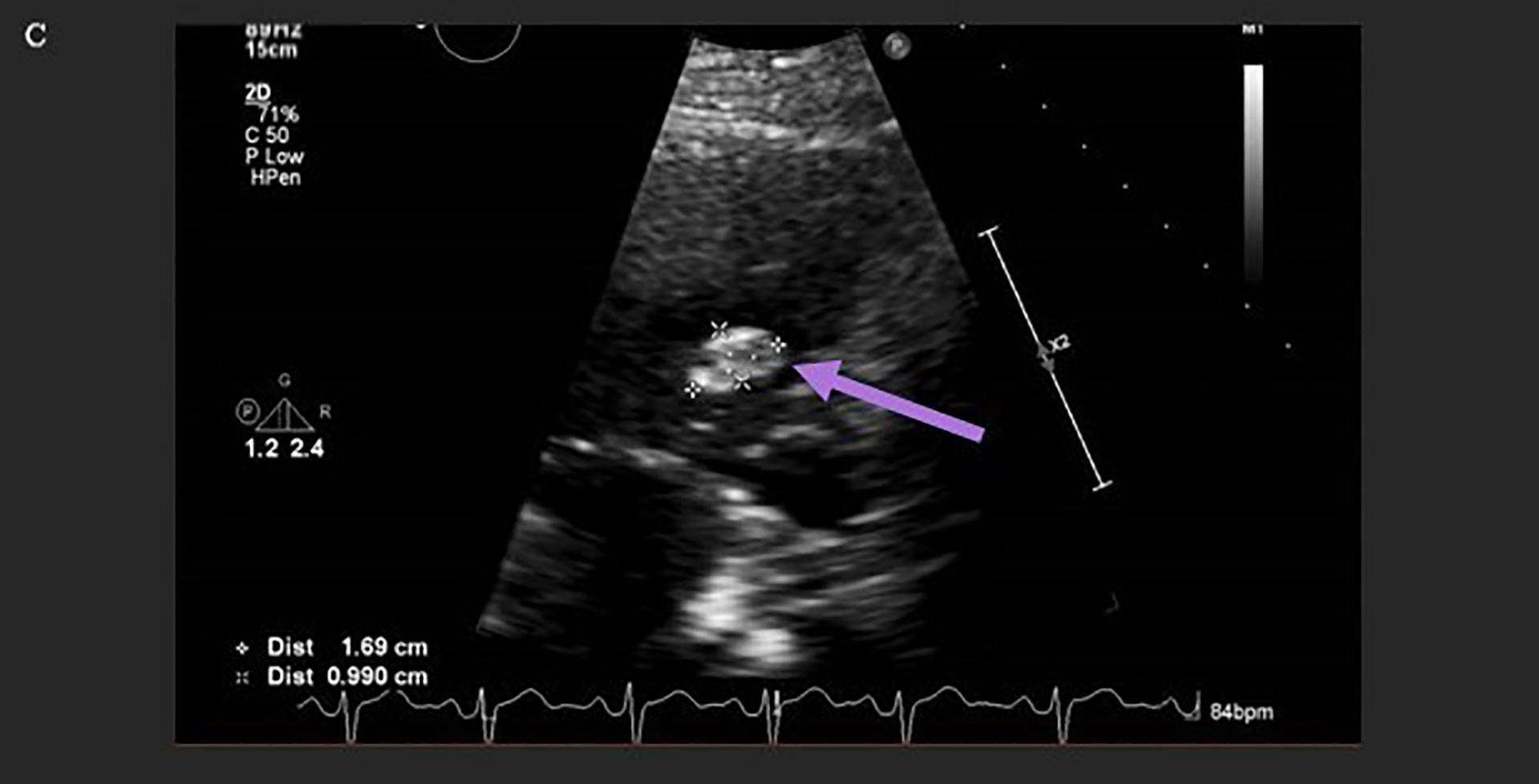

Supplement: Supplementary file 7 [file JETem-7-2-V14supp7.jpg]

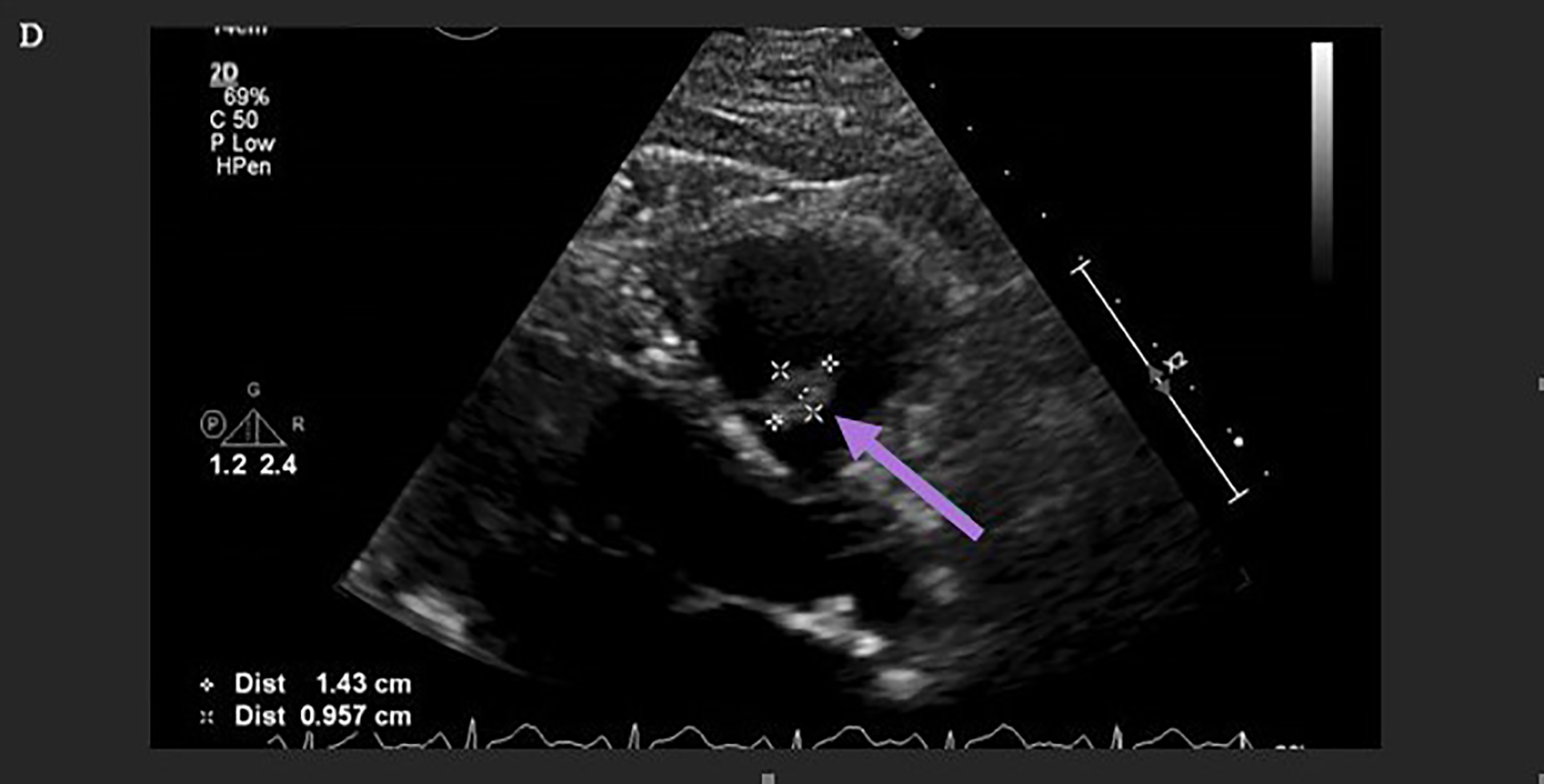

Supplement: Supplementary file 8 [file JETem-7-2-V14supp8.jpg]
